# Setting the IMPACT (IMProve Access to Clinical Trial data) Observatory baseline

**DOI:** 10.11613/BM.2018.010201

**Published:** 2018-02-15

**Authors:** Mersiha Mahmić-Kaknjo, Josip Šimić, Karmela Krleža-Jerić

**Affiliations:** 1 Department of Clinical Pharmacology, Zenica Cantonal Hospital, Zenica, Bosnia and Herzegovina; 2 Faculty of Medicine, University of Zenica, Zenica, Bosnia and Herzegovina; 3 Health Sciences Library, Faculty of Health Studies, University of Mostar, Mostar, Bosnia and Herzegovina; 4 IMPACT Observatory, Montreal, Canada; 5 Mediterranean Institute for Life Sciences - MedILS, Split, Croatia; 6 Croatian Cochrane Centre, Split, Croatia

**Keywords:** clinical trial data sharing, baseline, databases, registries, Cochrane, scandals

## Abstract

**Introduction:**

The aim of the IMPACT (IMProving Access to Clinical Trial data) Observatory is to assess the transformation of clinical trials (CT) related to the evolution of sharing of CT data. The objective of this study is to establish a baseline for monitoring CT data sharing by the Observatory.

**Materials and methods:**

In this scoping review we searched for publications that address sharing, dissemination, transparency or reuse of CT data published prior to December 31st 2000. Two authors screened titles and abstracts of 1204 records received by Medline searches and added 47 publications from direct discovery. Four researchers extracted, coded, and analyzed the predefined information from 102 selected papers.

**Results:**

We found a growing recognition of the importance of data sharing prior to 2001. However, there were numerous obstacles including the ambiguity of the concept of data sharing, the absence of specific terminology and the lack of an “open” culture. By the end of 2000, data, metadata, and evidence based medicine were defined. Data sharing, registries, databases and re-analyses of individual patient data (IPD) emerged. The use of systematic reviews and IPD meta-analysis in decision making was promoted. Most arguments for broader data sharing came from oncology, paediatrics, rare diseases, AIDS, pregnancy, perinatal medicine, and media reporting related scandals.

**Conclusions:**

Our findings indicate that the year 2000 could be used as a baseline for monitoring the evolution of CT data sharing as basic prerequisites were set in place, including greater understanding that CT data sharing is essential for decision making and the advancements of the Internet.

## Introduction

A clinical trial (CT) is a prospective controlled or uncontrolled study evaluating the effects of one or more health-related interventions assigned to human participants. In this paper, we use the term data sharing to describe the practice of making data from primary research publicly available for reuse. Many different types of data may be shared, including raw or analyzable data set; metadata, or “data about the data” (*e.g*., protocol, statistical analysis plan, and analytic code); and aggregate, summary-level data (*e.g.*, summary-level results posted in registries, lay summaries, publications, and clinical study reports) (*1*). Raw data, participant-level data and individual-participant data (IPD) are unprocessed data from a clinical trial which come in their original form (before the information has been analyzed or statistically manipulated) in contrast to aggregate data. They could be records of original observations, measurements, and health-related interventions, researcher’s records on patients, medical charts, hospital records, lab notes, evaluations, data recorded by instruments, attending physician notes, *etc.* (*2*).

Results from health research are often considered a public good, and data sharing seen as beneficial, particularly because the re-analysis of data is the basis of reproducible research, which can help better understand results of a trial and serve as the basis of pooling data from multiple trials, thus revealing new information beyond information gained from any single study (*3*-*5*).

Also, CT data sharing has been identified as useful to explain disagreements between individual CTs and prevent biases (*6*-*8*).

The objective of the IMPACT (IMProving Access to Clinical Trial data) Observatory is to assess the transition of clinical trials regarding data sharing due to ongoing initiatives, identify facilitators and barriers of clinical trials data sharing, indicate trends, and inform the process (*9*, *10*). As we needed to establish a baseline from which to start monitoring changes regarding data sharing we decided to perform a scoping review. Specifically, as one of IMPACT Observatory studies, this scoping review aims to explore to what extent CT data were shared prior to 2001 and to determine the appropriateness of setting the year 2000 as the baseline from which the IMPACT Observatory could start monitoring changes regarding data sharing (*10*).

Observatories or natural experiments are epidemiological studies that assess the impact of one or more interventions that are not controlled by the observatory researcher(s) to inform the process and indicate trends (*11*, *12*).

## Materials and methods

We performed a scoping review of the literature. A scoping review is a method used to better understand a phenomenon; it generally consists of mapping literature on a specific topic, and identifying key concepts, theories, and sources of evidence. It is particularly useful when a research question is broad and the goal is to identify qualitative rather than quantitative parameters (*13*, *14*). Literature searches were performed in Medline by two librarians using 8 different search strategies that were developed jointly with one reviewer. Searches were performed with no language limitations using strategies to select articles published prior to 2001 (*i.e.* up to December 31st 2000).

**Figure 1:**
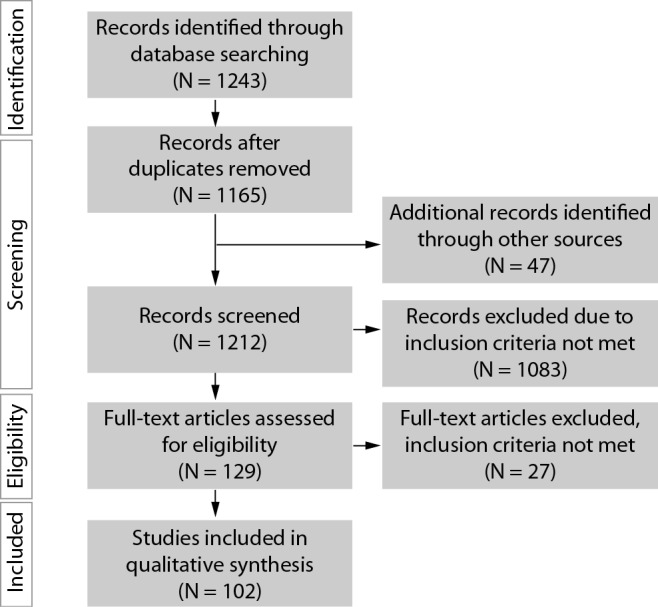
Flowchart of selection process

A flowchart of the selection process is presented in Figure 1. The following (MESH) terms were used: “clinical trials as topic“, ”information dissemination“, ”information storage and retrieval“, ”access to information“, ”disclosure“, ”drug industry, policy“ and looking for specific terms including “clinical trials dissemination storage and retrieval“, ”clinical trial, information dissemination“, ”dissemination policy“, ”clinical trial dissemination–drug industry“, ”disclosure” that included “drug industry”, and “access” that included “databases” and “policy”.

These searches yielded 1204 records. After deduplication and exclusion of papers published after December 31st 2000, titles and abstracts (if available) were screened by one reviewer, and then cross-checked by another reviewer. Inclusion criteria were as follows: any article reporting or possibly reporting on CT data sharing, databases, registries, repositories, re-analysis and related practice and/or policies published prior to December 31st 2000.

We also included 47 papers identified through previous work which met our inclusion criteria. Two of the authors independently evaluated 129 full text records for final inclusion. Upon full text assessment, 27 records were excluded as they did not meet inclusion criteria, but described patient registries, or librarian research, or sharing data other than the ones from clinical trials. Most discrepancies were solved by discussion; remaining disputes were resolved by the third author.

We analyzed a total of 102 full texts. Two of the authors independently extracted relevant information in a predefined Excel file (Microsoft, Redmond, USA). A different pair of authors eliminated duplicates and summarized the information. We analysed two groups of topics: one group of topics (headings) in the Excel were phenomena including data sharing, database, registries, repository. The other group of topics (heading) were disease and/or patient groups and scandals related to data sharing. This group of headings was further expanded to capture specific disease or patient groups, as the preliminary analysis indicated that they appeared multiple times and created the atmosphere or even directly called for data sharing, for transparency.

All three authors coded the information for the following topics: data sharing, registry, databases, oncology, AIDS, pregnancy and perinatal medicine, child health/rare diseases, adverse effects, re-analysis, fraud/falsifications, scandals, individual patient/participant data (IPD), publication bias.

## Results

**Figure 2:**
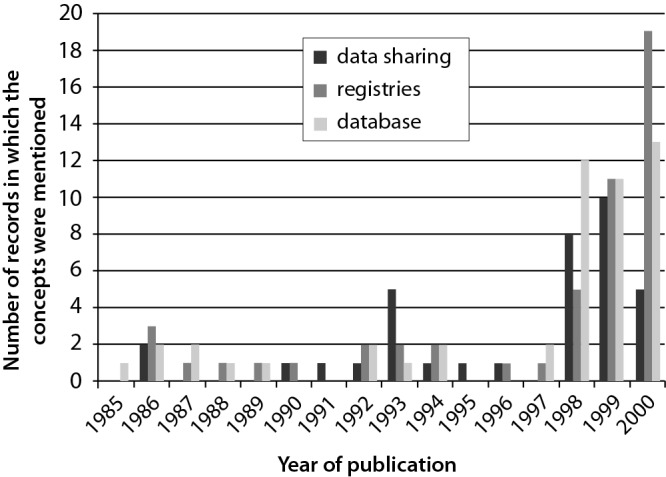
Number of records addressing data sharing, registries, and database in selected literature prior to December 31st 2000

In our analysis of the 102 selected papers, we identified 3 major concepts of interest: data sharing, registries and databases. Figure 2 illustrates that this discussion peaked in years 1986 and 1993, however, significant expansion in the volume of relevant literature occurred between 1998 and 2000.

**Figure 3:**
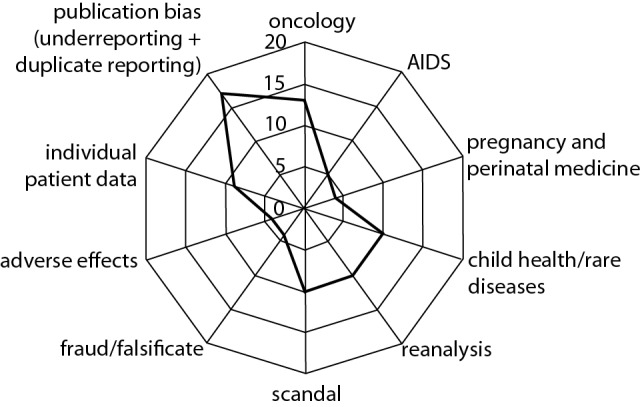
Data sharing by topic and health area in which data sharing is addressed in the literature published prior to December 31st 2000.

As can be seen on Figure 3, calls for data sharing came from several health areas, most frequently from oncology, followed by child health/rare diseases, AIDS, and pregnancy and perinatal medicine. The most frequent topic discussed was publication bias, including both underreporting and duplicate reporting.

However, the terminology was very ill-defined. For example, the term “database” was used extensively but described the collection of very diverse records, including bibliographic databases (PubMed, EMBASE, *etc.*). Also, different terms were used to describe similar activities or similar systems. The term “registry” was used for observational databases, clinical registries, patient registries and disease oriented registries; “trial banks” were also called large population cohorts, administrative databases, electronic patient records systems, large-scale databases, and databases of hospital records (*15*, *16*). In the nineties, the term registry was introduced to refer to a collection of data from CT protocols (*17*-*22*). The term “individual patient data” was used either to indicate data collected as a part of providing routine healthcare for an individual patient, or data collected during a prospective clinical trial. The acronym IPD was used to indicate the Individual Patient Data, while now, in case of clinical trial IPDs, it stands for Individual Participant Data (*23*, *24*).

**Figure 4:**
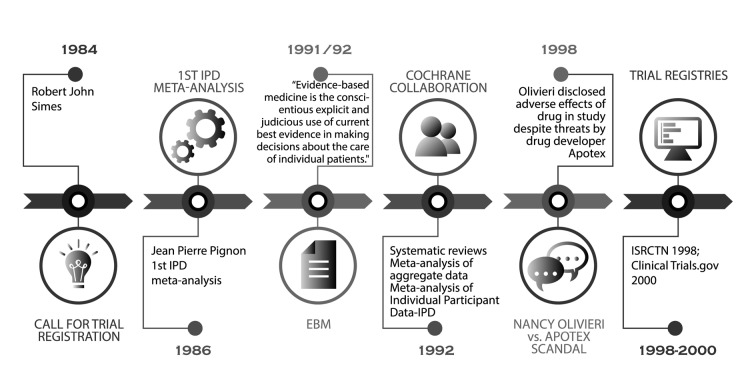
Key milestones of clinical trial data sharing prior to 2001. EBM – evidence based medicine. IPD – individual patient/participant data.

We selected actions and events that had major impact on the evolution of CT data sharing prior to 2001 and presented them in Figure 4. These key milestones include the call for trial registration and the establishment of CT registries (International Standard Randomized Controlled Trial Number (ISRCTN) registry and Clinicaltrials.gov), IPD meta-analyses, the onset of the Cochrane Collaboration and the Evidence Based Medicine (EBM) (*19*-*22*, *24*-*28*). We also included Nancy Olivieri scandal as it influenced the practice of industry - academia functioning (*29*, *30*).

We also selected highlights of discussion of the evolution of data sharing and presented them in the Table 1. These include citations of *pros* and *cons* arguments, as well as some actions like starting of databases and studies using data from industry sources.

**Table 1:** Highlights of discussion of data sharing evolution prior to December 31st 2000

**Year**	**Event or citation**	**Reference**
1982	Launch of the Physician Database Query - an online database that provides summaries of current ongoing research treatment protocols (1000) directly supported by the NCI; summaries of all active clinical trials shared	(*35*) (*36*)
1982	National Cancer Institute started a database of clinical trials and cancer treatment; summaries of 1100 protocols were shared as of 1988, as well as contact details of participants - name(s), address(es), and telephone number(s)	(*17*)
1987	The launching of KIGS international outcomes research database of longitudinal data on growth hormone therapy in children and adults. Of note, individual physicians retain the right to use their patients’ data for their own research purposes	(*18*)
1992	„Freedom of access and the sharing of research results have, traditionally, been values of science... The objectivity, accuracy, and reliability of observations and data interpretation that science requires can be destroyed subtly by financial incentives and by institutional and personal pride... Sharing of information is seriously impeded because investigators are in competition with one another for the market. “	(*39*)
1993	“ ... Individual companies may accept the principle that data from their clinical trials are a public as well as a private resource that needs to be made available for review...The pressure of publicity may achieve something. “	(*40*)
1993	“Large datasets can yield worthwhile information... At present, manufacturers guard the results of their in-house studies: they must be persuaded to be more open. Patients and physicians should insist that data derived from their care be made available (with full confidentiality protected) for other physicians to draw on.”	(*6*)
1993	There are no clear-cut rules governing access by other scientists to clinical trials’ data. The notion of routine archiving of clinical trials’ data is still fairly new.	(*41*)
1994	“No one would expect investigators to publish their raw data.”	(*31*)
1994	“Unlocking large datasets is not reliable in this respect and is certainly not cheap.”	(*38*)
1995	Cochrane working group on meta-analysis using individual patient data issued „Practical methodology of meta-analyses (overviews) using updated individual patient data“	(*23*)
1996	„Trial and error are still the fundamentals of advancement...The key to the sensible handling of data is a careful interpretation of observed associations rather than the artificial erection of barriers. “	(*8*)
1998	Repository first mentioned as a term for a database that wold enable virtual clinical trials, by a “ comprehensive, integrated view of the diverse population cared for by the University of Pennsylvania Health System”.	(*42*)
1998	A group of authors of a meta-analysis contacted manufacturers regarding other unpublished and published randomized controlled trials,but the authors specified no other details.	(*43*)
1998	Individual studies from Abbott Laboratories’ clinical database are accessible for re-analysis.	(*44*)
1998	„All trial evidence should be published before new drugs are marketed, and medical journals should not carry advertisements referring to unpublished data. “	(*32*)
1998	„Paramount in the new ethics of epidemiological research is concern for the place of individual informed consent to the disclosure and use of personal information maintained in proprietary databases, governmental registries, and medical records.“	(*45*)
1998	ISRCTN Clinical trial registry started. In response to the growing body of opinion in favor of prospective registration of randomized trials, the Science Navigation Group launched the CCT website in 1998 as the metaRegister of Controlled Trials, mRCT. In 2003 it added an unique ID to each trial and renamed it to ISRCTN. It is possible to cite a trial using this ID number.	(*22*)
1999	„Information about published and unpublished clinical trials should be ideally placed in a registry. Internet communication will be important mean of exchange of information.“ Stated R. Horton and R. Smith, editors of Lancet.	(*37*)
1999	Anonymised data (sociodemographic data without revealing a patient’s identity: employer and insurer names, race, ethnicity, and age) in the cancer outcome database.	(*46*)
**Year**	**Event or citation**	**Reference**
1999	“The request to enroll and file a CRF will be sent to the clinical trials repository. The repository will interpret the request and generate a dynamic CRF for the user to fill out. Corrections will be completed at the time of filing, according to specifications in the repository, and the “clean” CRF will be returned to the repository.”	(*47*)
1999	The manufacturer (Pfizer) provided some unpublished information from clinical trials, mentioned as a reference „Data on file“	(*48*)
1999	A comprehensive data file of the manufacturer (no details) utilized to evaluate the clinical safety profile of a drug.	(*49*)
1999	Use of patient data from observational databases (EuroSIDA, the French Hospital Database on HIV and the Swiss HIV) to evaluate the effectiveness of antiretroviral therapy for HIV infection: comparison of cohort studies with randomized trials.	(*50*)
2000	The manufacturer (Novo Nordisk) provided unpublished information, mentioned as a reference (Data on file).	(*51*)
2000	Large complex databases are available to scientists, information stored and can be tested using this well of knowledge	(*33*)
2000	ClinicalTrials.gov established in February 2000. Contained information about approximately 5000 trials.	(*20*)
2000	„In January 2001, the United Kingdom will become the first place to offer public access to a database of all UK clinical trials on new medicines sponsored by the pharmaceutical industry“ announced the Association of British Pharmaceutical Industry (ABPI).	(*52*)
2000	„Now that electronic publication is possible there is no reason why every well-conducted clinical trial is not published.” Commented Iain Chalmers, director of the UK Cochrane centre.	(*52*)
NCI - National Cancer Institute. KIGS - Kabi Pharmacia & Upjohn International Growth Database. ISRCTN - International Standard Randomized Controlled Trial Number. CCT -Current Controlled Trials. CRF - Case Report Form. EuroSIDA - Pan-European Observational Study of HIV infected patients.		

As can be seen in the Table 1, the last two decades of 20^th^ century are rich in actions and discussion regarding sharing and reuse of CT data. Related discussions considered both the advantages and disadvantages or risks of these practices.

In 1992 Chalmers *et al.* identified weaknesses in all stages that took place between research and practice, from design and conduct of clinical trials, to the use of their results in decision making (*25*). In 1992, Pignon *et al.* published the first meta-analysis in which raw data were used (*24*). The Evidence Based Medicine started to form (*26*-*28*). Several important reasons for sharing raw data were put forward in the literature: the inclusion of unpublished data in meta-analyses could decrease publication bias, and improve the relevance of the question, the interpretation of results, and the design of future trials, as well as help avoid unnecessary duplication of research (*24*, *25*, *31*-*33*).

During this period, it was acknowledged that more powerful analyses could be run, stratified by trials, including subgroup analyses made possible if IPD data were available (*24*). The importance of IPD meta-analysis to produce evidence, develop guidelines, and support decision-making was increasingly discussed (*23*, *24*, *34*).

In 1995, the Cochrane Collaboration convened a workshop in London, UK, to discuss the practicalities of meta-analyses based on IPDs (*23*). As it became clearer in 1996, that data could and should be reused, Michels and Rosner concluded that it is more important to raise awareness on sensible handling of data than to artificially erect the barriers to data sharing (*8*). The importance of sharing raw data was further promoted by Vamvakas and Blajchman who noted that raw data meta-analysis could solve disagreements between individual randomised controlled trials (RCTs) (*7*).

During this decade, there also were breakthrough developments in the area of information technology, both in terms of software and hardware, which provided the underlying means to store and manage information (*35*, *36*). The development of the Internet was quickly recognized as an important tool for sharing growing corpus of information, such as raw data (*33*, *37*).

## Discussion

Our scoping review reveals that CT data sharing intensified during the last two decades of 20th century, helping forge a strong consensus that a strict baseline could not be drawn, but the real deal breaker was using individual patient data in a meta-analysis in 1992 (*24*). In this dynamic process a key terminology was defined, and new methodologies were pioneered. This process was led by the Cochrane Collaboration, which sought to improve the quality of systematic reviews and of the IPD meta-analysis, and developed and published methodological guidance for IPD meta-analysis (*23*). There was a broad discussion on the benefits of data sharing, often led by HIV/AIDS or cancer patient groups, as well as media reports related to the harmful consequences of not sharing of CT data. However, the growing consensus related to the value of data sharing was matched by numerous obstacles including the lack of data sharing culture, ambiguity surrounding key data sharing concepts and terms, some of which we described in this paper. Perceptions about data sharing wobbled between *pros* and *cons*. For example, negative attitudes towards data sharing were expressed in mid-nineties: Glass was convinced that „no one would expect investigators to publish their raw data” (*31*). McCarthy argued that datasets should be available to both physicians and patients, while Warlow *et al.* argued that unlocking large datasets is neither reliable nor cheap (*6*, *38*). Gradually towards the end of the century data sharing was increasingly seen as a benefit, although practical solutions needed to be implemented to facilitate the practice. One possible explanation for this evolution could be the newly founded Cochrane Collaboration, which promoted sharing of IPD and its use to conduct meta-analyses and also the creation of the concept of the EBM (*23*, *26*-*28*).

To the best of our knowledge, this early stage of clinical trial data sharing has never been analyzed, so this paper might contribute to understanding of the process, with its systematic design as the most important strength.

The major limitation of our study is a lack of specific MESH terms for our scoping review. Furthermore, literature searches were performed only in Medline. In order to compensate for these limitations, two librarians performed searches independently and we added references we discovered through other related work.

## Conclusions

Throughout the last two decades of 20th century we observed glimpses and hints of attempts of CT data sharing and gradual emerging of the awareness of risks and benefits related to data sharing. Multiple factors contributed to this evolution. Public media and scientific journals played a significant role in raising awareness and influencing the change of culture regarding CT data sharing. Vulnerable populations (cancer patients, AIDS patients, pregnant women, children, rare diseases, expensive therapies) frequently participated in breakthrough cases.

At the end of the century, the basis for further development was laid and the year 2000 ends with initial trial registries, definition of datasets, the Cochrane Collaboration, enhanced systematic reviews and emerging IPD meta-analysis, the use of evidence gained by IPD meta-analysis for development of clinical guidelines and for making decisions that would benefit patients, constant improvement of Internet features, all in the environment of the growing interest, pressure and discussion about the need for data sharing by various constituencies.
